# East Learns from West: Asiatic Honeybees Can Understand Dance Language of European Honeybees

**DOI:** 10.1371/journal.pone.0002365

**Published:** 2008-06-04

**Authors:** Songkun Su, Fang Cai, Aung Si, Shaowu Zhang, Jürgen Tautz, Shenglu Chen

**Affiliations:** 1 College of Animal Sciences, Zhejiang University, Hangzhou, China; 2 Centre of Excellence in Vision Science, Research School of Biological Sciences, The Australian National University, Canberra, Australia; 3 BEEgroup, Biocenter, University of Würzburg, Am Hubland, Würzburg, Germany; Centre de Recherches su la Cognition Animale - Centre National de la Recherche Scientifique and Université Paul Sabatier, France

## Abstract

The honeybee waggle dance, through which foragers advertise the existence and location of a food source to their hive mates, is acknowledged as the only known form of symbolic communication in an invertebrate. However, the suggestion, that different species of honeybee might possess distinct ‘dialects’ of the waggle dance, remains controversial. Furthermore, it remains unclear whether different species of honeybee can learn from and communicate with each other. This study reports experiments using a mixed-species colony that is composed of the Asiatic bee *Apis cerana cerana* (*Acc*), and the European bee *Apis mellifera ligustica* (*Aml*). Using video recordings made at an observation hive, we first confirm that *Acc* and *Aml* have significantly different dance dialects, even when made to forage in identical environments. When reared in the same colony, these two species are able to communicate with each other: *Acc* foragers could decode the dances of *Aml* to successfully locate an indicated food source. We believe that this is the first report of successful symbolic communication between two honeybee species; our study hints at the possibility of social learning between the two honeybee species, and at the existence of a learning component in the honeybee dance language.

## Introduction

When foraging honeybees find an attractive food source, they can perform a special communicative behaviour called the dance language, which was first discovered by Karl von Frisch [1]. Dances can be classified into three broad forms [1] which depend on the distance of the food source. For sources close to the colony, a simple round dance is performed. For larger distances, a sickle dance is performed. Finally, at the furthest distances from the nest, a waggle dance is performed. The waggle dance is the most sophisticated of these three forms as it encodes direction and distance of the food source [1]. Each iteration of the honeybee waggle dance consists of a straight waggle phase, whose duration indicates distance to the food source, and whose direction relative to gravity encodes the direction of food relative to the sun's azimuth [1]–[3]. Distance to a food source is gauged through the optic flow experienced on the outbound foraging trip [4]–[6]. Recently, the waggle phase, instead of the entire circuit of the dance, was confirmed as a reliable indicator of the distance to the food source [3], [7]–[9]. By eavesdropping on this communication system, scientists have obtained a unique perspective into the perceptual world of insects [10].

Honeybee colonies achieve fitness through dancing to share food-location information among their nest mates [11]. The dance language of the honeybee is thought to have evolved from a more primitive form of communication, perhaps similar to that of extant bumblebees [12]. Moreover, various honeybee species may have evolved distinct ‘dialects’ during their long evolutionary history [13], [14]. Dance ‘dialect’ describes the distances at which foragers of each *Apis* subspecies make the transition between dance types. According to older published distance communication curves [15], [16], *Apis florea* and *Apis mellifera carnica* display striking differences in their dialects. Further research has shown that the dance language could be influenced and affected by both genetic factors [17]–[19] and environmental parameters [4]–[6]. Some comparative studies, based on these later findings, have shown that *Apis mellifera carnica* and *Apis florea* do not differ significantly from each other in the waggle phases performed as a function of distance [20]. However, these published waggle curves of different honeybee species were neither obtained from the same spatial route, nor at the same time. Thus, the question of whether these differences are real, or simply the result of flying through dissimilar visual environments, remains unanswered.

It is therefore necessary to obtain waggle curves of different species made to forage in the same location and at the same time. A mixed-species study on dance language communication is one possible way to investigate this issue. In general, individual honeybees from different species cannot be put together in one colony, because they have their own special odour, and are likely to attack and kill each other quickly [21]. This is the main obstacle to the study of social learning and communication between different species of *Apis*. Although honeybee workers and queens can be reared in any single-species colony, interspecific reciprocal introductions of female larvae between *Apis mellifera* and *Apis cerana* have usually failed [22], [23], because of species-specific brood pheromones [23], [24] and/or differences in royal jelly [25], [26]. However, encouraged by the reports that young European *Apis mellifera* workers are accepted into Asian *Apis cerana* colonies [27]–[29], we assembled a harmonious mixed-species colony of *Apis cerana cerana* and *Apis mellifera ligustica*. Using this mixed-species colony, we were able to investigate the communication and learning of the dance language between individuals of different honeybee species.

## Results

### Successful establishment of a mixed-species colony

**Figure 1:**
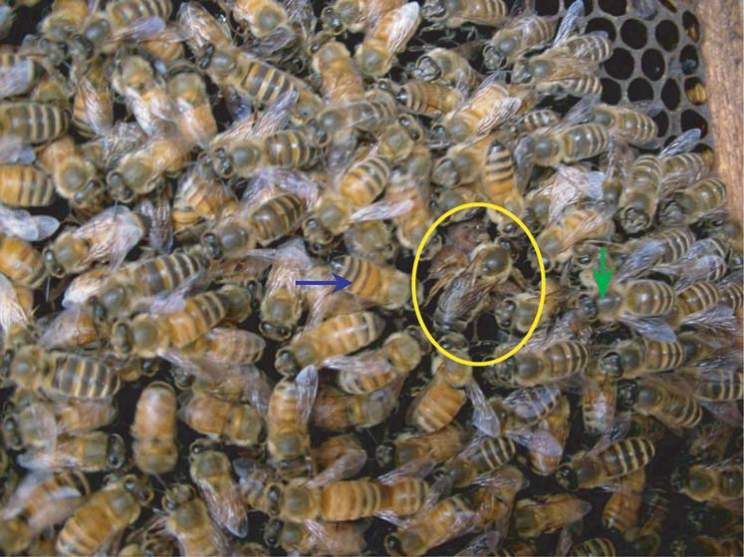
The mixed-species colony with an *A. cerana cerana* queen, *A. cerana cerana* workers and *A. mellifera ligustica* workers. The yellow circle indicates the *Acc* queen. Blue and green arrows indicate *Aml* and *Acc* workers, respectively. The mixed-species colony was organized as follows: we put two sealed *Aml* brood frames with about 5,000 pupae into a healthy *Acc* colony containing two frames, honey, pollen, brood, ∼5,000 workers and one queen. The mixed-species colony had around 5,000 workers each of *Acc* and *Aml* after 12 days.

We organized two types of mixed colonies consisting of an *Apis cerana cerana* (*Acc*) queen, *Acc* workers and *Apis mellifera ligustica* (*Aml*) workers, and two other mixed colonies consisting of an *Aml* queen, *Aml* workers and *Acc* workers. In the former colonies, the workers cohabited well for more than 20 days, while in the latter colonies, the *Acc* workers were killed and cleaned up by *Aml* workers after 2–3 days. Thus, we were only able to use the former mixed-species colony to carry out our experiments (see figure 1). We put the mixed colonies into observation hives when we transported them to the experimental location.

As stated previously, the bees were able to coexist harmoniously in the mixed-species colonies with pre-existing *Acc* adults. With the maturation of *Aml* workers, however, the mixed colonies sometimes became unstable when special tasks such as cleaning were required. Some hostile individuals began to attack and bite the other species' individuals. The hostile activities could become more and more intense over the following 1–2 days, until all the *Aml* individuals were attacked and removed from the mixed-species colony. Once such episode of instability became a serious obstacle for the successful completion of our experiments. We took several measures, including feeding the mixed colonies sugar syrup at the night, spraying honey water on the surface of the honeycomb when it was in turmoil, and removing the more aggressive individuals, to make the mixed-species colony harmonious. These measures proved to be very successful in modulating the temperament of the mixed-species colony during periods of stress such as shortage of food, bad weather or cleaning. This indicates that the threshold for discriminating between individuals of different species could be changed by altering the colony's social and physical conditions. We were thus able to manage the mixed-species colony very well throughout the experimental period, and both species of honeybees lived harmoniously in the same colony for more than 50 days (Movie S1).

### *Acc* and *Aml* possess distinct ‘dialects’ of the waggle dance

**Figure 2:**
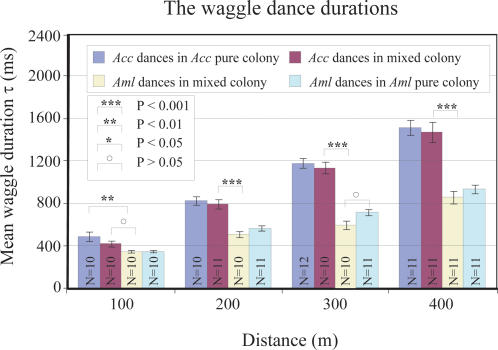
Waggle dance durations of *Apis cerana cerana* (*Acc*) and *Apis mellifera ligustica* (*Aml*) in single-species and mixed colonies. Blue bars - waggle duration of *Acc* in the *Acc* pure colony; purple bars - waggle duration of *Acc* in the mixed-species colony; yellow bars - waggle duration of *Aml* in the mixed-species colony; and green bars - waggle duration of *Aml* in the *Aml* pure colony. N in each bar denotes number of individual. *** denotes statistically significant difference at p<0.001, ** denotes p<0.01, * denotes p<0.05 and ο denotes p>0.05. *Acc* waggle durations in the mixed hive are significantly higher than those of *Aml* for all feeder positions. Dances in the mixed hive are not significantly different from dances in the single-species hives for either species.

**Figure 3:**
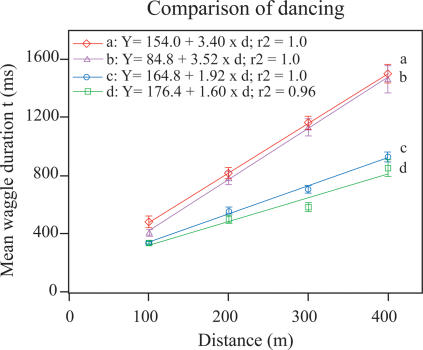
Distance calibration curves of *A. cerana cerana* and *A. mellifera ligustica.* a: *Acc* in the *Acc* pure colony; b: *Acc* in the mixed-species colony; c: *Aml* in the *Aml* pure colony; d: *Aml* in the mixed-species colony. There is no significant difference between the linear regression lines a and b (p>0.05) or c and d (p>0.10), but there is a significant difference between a and c (p<0.001) and b and d (p<0.01) (see text for details).

Figure 2 shows the waggle dance duration of *Aml* and *Acc* foragers, from mixed or pure colonies, that had been trained to an artificial feeder placed at different distances (100, 200, 300 and 400 m away). Under normal conditions, *i.e.* in single-species colonies, *Acc* foragers consistently had a much greater waggle duration for a given distance than did *Aml* foragers, as has been previously reported [13]. Further, the waggle durations of dancers from both species increased in a linear manner with increasing distance. The slope of the distance-waggle duration curve for *Acc* in the single-species colony was significantly steeper than that of *Aml* bees in the single-species colony (pairwise comparison, t-test, t = 8.8, d.f. = 6, P<0.001, Figure 3). The slope for *Acc* in the mixed-species colony was also significantly steeper than that of the *Aml* bees in the same colony (pairwise comparison, t-test, t = 4.4, d.f. = 6, P<0.01, Figure 3). Moreover, at the 100, 200, 300 and 400 meter positions, foragers of a particular species generally displayed similar waggle durations, regardless of whether they were from a single-species or mixed-species colony (P>0.05, ANOVA for Two-stage Nested Design and Tukey's test, see Table S1). The slope of the distance-waggle duration curve for *Acc* in the single-species colony was not significantly different to that of *Acc* in the mixed-species colony (pairwise comparison, t-test, t = 2.3, d.f. = 6, P>0.05, Figure 3). Similarly, the curve for *Aml* in the single-species colony was not significantly different to that of *Aml* in the mixed-species colony (pairwise comparison, t-test, t = 1.44, d.f. = 6, P>0.05, Figure 3). The waggle duration results show that there really are dialect differences between *Acc* and *Aml*. The mean dance angle, the direction of the waggle run relative to the vertical direction, was not significantly different (P>0.05, pairwise t-test) between *Acc* and *Aml* in the mixed-species colony (Table 1; see Methods for details of analysis).

**Table 1:** Dance angles comparison between *Acc* and *Aml* in mixed-species colony

Positions	100 meter				200 meter				300 meter				400 meter			
	IN	DA	IN	DA	IN	DA	IN	DA	IN	DA	IN	DA	IN	DA	IN	DA
**Ten pairs of dance angles**	Acc20	41.8	Aml12	41.4	Acc042	55.8	Aml01	56.4	Acc20	28.6	Aml34	27.2	Acc414	−43.4	Aml444	−42.8
	Acc23	35.2	Aml01	35.8	Acc213	−14.4	Aml32	−12.4	Acc344	7.4	Aml34	5.4	Acc344	10.6	Aml23	13.2
	Acc32	36.4	Aml22	32.4	Acc213	−0.8	Aml041	−0.8	Acc14	9.8	Aml14	13.6	Acc042	12.6	Aml041	12.4
	Acc01	34	Aml02	31.6	Acc213	−2	Aml12	−1.6	Acc10	−53	Aml21	−47.6	Acc344	27.6	Aml02	28.4
	Acc04	31	Aml30	31.6	Acc144	36.6	Aml01	35.8	Acc20	−42.2	Aml113	−40	Acc40	−47.2	Aml111	−50.4
	Acc23	33.8	Aml20	37.4	Acc224	20.6	Aml444	18	Acc34	−45.6	Aml113	−40.2	Acc344	17.6	Aml33	18.8
	Acc23	32.6	Aml12	36.4	Acc233	17.8	Aml31	18.4	Acc111	−40	Aml113	−41.6	Acc344	19.4	Aml23	15.2
	Acc32	36	Aml30	35.2	Acc014	17.2	Aml444	18.6	Acc111	−43.4	Aml113	−39	Acc223	16.6	Aml041	15.6
	Acc20	33.8	Aml31	36.4	Acc14	18.2	Aml31	17.6	Acc10	−41.4	Aml113	−40.6	Acc124	11.2	Aml33	11.4
	Acc01	33.6	Aml21	36	Acc12	17.4	Aml041	15.4	Acc111	−43.4	Aml01	−41.2	Acc20	14.6	Aml44	12.4
**Mean±SD**	34.82±2.09		34.45±2.14		16.64±14.24		16.54±13.98		−26.32±21.05		−24.4±20.06		3.96±18.91		3.42±19.32	
**T-test**	P = 0.4797, df = 9, t = 0.7373				P = 0.8304, df = 9, t = 0.2206				P = 0.0634, df = 9, t = 2.1161				P = 0.4393, df = 9, t = 0.8092			

Note: IN individual number; DA dance angles. The dance angle determined with the method in text. We use partnership T-test of DPS statistical software to analyse the data. There were no significant differences in the dance angles between *Acc* and *Aml* in the mixed-species colony at any positions.

### *Acc* foragers can be recruited by *Aml* dancers in a mixed-species hive

In such a hive, we found that both *Acc* and *Aml* workers followed the dances of *Aml* and *Acc* foragers (see Supporting Information Movie S2 and S3). In the first recruitment experiments, ten different individuals of *Acc* were recruited by *Aml* dancers to a feeder that was located at either 50, 100, 150 or 200 m, and marked with distinctive paint marks. At the same time, there was another (control) *Acc* colony 5 meters away from the observation hive position. We monitored the entrances of both colonies when the foragers came back from the feeder. We did not find a single *Acc* forager from the feeder at this control colony, indicating that the *Acc* bees at the feeder had been recruited, and had not merely found it through random searching.

In the second recruitment experiment, we were able to mark *Acc* workers while they were following *Aml* dances, as the observation hive was equipped with a movable glass cover. More than 20 followers were marked for each feeder position. An average of 19.5% (Table 2) of these marked *Acc* followers were found at each feeder over the next ten minutes to two hours, and were caught and held in a bottle for the remainder of the experiment. In both recruitment experiments, as there were no *Acc* dancers from the feeders, *Acc* foragers could only obtain the relevant foraging information from *Aml* foragers by decoding their dance language.

**Table 2:** *Acc* foragers recruited by *Aml* dancers

Feeder Positions (M)	50	100	150	200
**Number of marked ** ***Acc*** ** followers**	24	24	27	28
**Number of recruited ** ***Acc*** ** foragers**	5	5	5	5
**Percentage (%)**	20.8	20.8	18.5	17.9
**Mean±SD (%)**	19.5±2.42			

Note: At 50, 100, 150, 200 m feeder positions, the Acc followers were marked with coloured paint on their thorax and/or abdomen while they followed Aml dancers in the mixed-species hive.

These results support our assertion that *Acc* foragers could follow the dances of *Aml*, and use this information to successfully forage at particular feeder positions in a mixed-species colony. A critical issue in these experiments was the fact that only one feeder was present at a time. Thus, one could argue that when bees of one species were found at the right location, as advertised by the other species, it was not because they were able to decode the ‘foreign’ distance information, but simply because they had no alternative choice. In order to address this possible criticism, we performed further recruitment experiments in which recruited bees were offered different feeders at different locations simultaneously.

### Accuracy of recruitment success by *Aml* dancers

**Figure 4:**
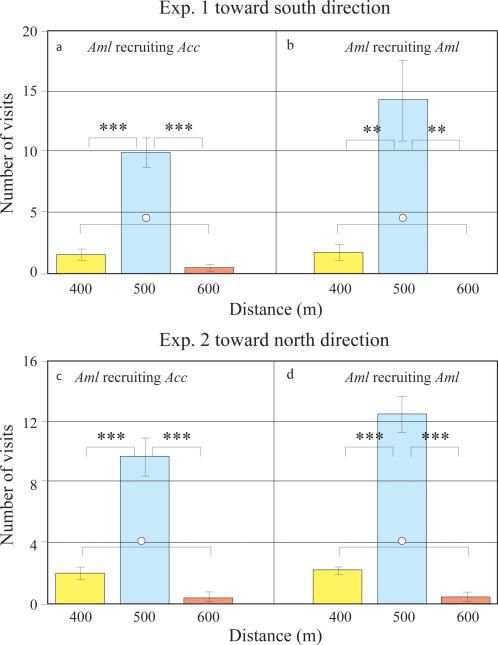
Accuracy of *Acc* and *Aml* foragers recruited by *Aml* dancers. Two experiments were carried out on the banks of the Da-Mei-canal in Zhangzhou, Fujian province, China. The results show that the vast majority of new recruits (both *Acc* and *Aml*) appeared or searched at the correct (500 m) position (See text for details).

To assess the accuracy of *Acc* foragers recruited by *Aml* dancers and vice versa, we carried out a group of complementary experiments on the banks of the Da-Mei-canal in Zhangzhou, Fujian province, China (see materials and method for details) in which recruiting bees were trained to a feeder located 500 m south-west from the hive. Recruited bees faced a choice between three feeders placed at 400, 500 and 600 m south-west from the hive, and aligned in the direction of the original feeder. Figure 4a shows the visiting numbers of *Acc* foragers recruited by *Aml* dancers at 400 m, 500 m and 600 m in south-west direction from the hive. The visit frequency of recruited *Acc* at 500 m was significantly greater than that at 400 m and 600 m (*F_2,9_ = *44.59, p = 0.0001, One-way ANOVA), and the frequency of recruited *Acc* at 500 m was greater than that at 400 m (P = 0.0001, Tukey's test) and 600 m (P = 0.0001, Tukey's test). Comparing visit frequencies at 400 m and 600 m, more bees visited the 400 m feeder than the 600 m feeder, but the difference was not significant (p = 0.6511, Tukey's test). Figure 4b shows the visit frequency of *Aml* foragers recruited by *Aml* dancers. We can see that the results are similar to those presented in Figure 4a (*F_1,24_ = *0.0402, p = 0.8509, ANOVA for Two-stage Nested Design). Once again, the visit frequency of recruited *Aml* was significant different at 400 m, 500 m and 600 m (*F_2,9_ = *15.813, p = 0.0011, One-way ANOVA), and the frequency of recruited *Aml* at 500 m was greater than that at 400 m (P = 0.0038, Tukey's test) and 600 m (P = 0.0016, Tukey's test). Comparing visit frequencies at 400 m and 600 m, more bees visited the 400 m feeder than the 600 m feeder, but the difference was not significant (p = 0.8061, Tukey's test). Comparison of *Acc* foragers and *Aml* foragers recruited by *Aml* shows that *Aml* dancers can recruit slightly more *Aml* foragers to the 500 m feeder than *Acc* foragers, but there was no significant difference (*F_1,6_ = *7.375, p = 0.135, One-way ANOVA).

In the second experiment, three identical feeder-stations were located along the Da-Mei canal, in the north-east direction from the hive, at 400 m, 500 m and 600 m respectively. The methodology was similar to the first experiment, and all the bees used in the previous experiment were captured at the start. The results of the second experiment confirmed the results of the first experiment. The visit frequency of recruited *Acc* was also significantly different among 400 m, 500 m and 600 m as shown in Figure 4c (*F_2,9_ = *40.79, p = 0.0001, One-way ANOVA). The frequency of recruited *Acc* at 500 m was greater than that at 400 m (P = 0.0002, Tukey's test) and 600 m (P = 0.0001, Tukey's test), but the visit frequency of recruited *Acc* at 400 m was not significantly greater than that at 600 m (p = 0.3979, Tukey's test). Figure 4d shows the same pattern for *Aml* foragers recruited by *Aml* dancers to the three feeders. The visit frequency of recruited *Aml* was significantly different at 400 m, 500 m and 600 m (*F_2,9_ = *80.68, p = 0.0001, One-way ANOVA), and the frequency of recruited *Aml* at 500 m was greater than that at 400 m (P = 0.0001, Tukey's test) and 600 m (P = 0.0001, Tukey's test), but the visit frequency of recruited *Aml* at 400 m was not significantly greater than that at 600 m (p = 0.2521, Tukey's test). The visit frequency of *Aml* foragers recruited by *Aml* dancers was similar to that of *Acc* foragers (*F_1,24_ = *0.0703, p = 0.804, ANOVA for Two-stage Nested Design).

In the third experiment, we partially repeated the above investigations, with the difference that *Acc* dancers were now made to recruit *Acc* and *Aml* foragers to a rewarded feeder flanked by two dummy feeders (Figure S1). The feeders were placed along a line in the north-east direction.

### *Acc* is able to acquire food-related information from *Aml* in the mixed hive

During the experimental period, trophallaxis was observed frequently between *Acc* and *Aml* workers (see Supporting Information Movie S4), which also shows that *Acc* and *Aml* could communicate food-related information with each other by transferring food reward.

### The number of *Acc* followers is greater than that of *Aml* followers in the mixed-species colony

**Figure 5:**
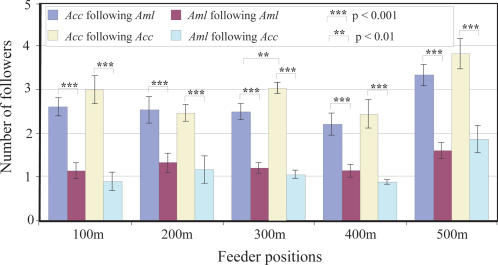
Comparison of follower numbers of *Acc* and *Aml* in the mixed-species colony. The number of *Acc* following *Aml* was significant larger than the number of *Aml* following *Aml* at all five positions. The number of *Acc* following *Acc* was also significantly larger than the number of *Aml* following *Acc* at all five positions. However, individuals of one species generally had a similar probability of following dancers of either species. The only exception is for *Acc* followers at the 300 m position.

Our observations of dance-following behaviour in the mixed-species colony indicate that *Acc* bees were much more likely to follow a dancer than *Aml* bees (P<0.001, t-test). However, individuals of each species generally displayed a similar likelihood of following dancers from both species (Figure 5).

## Discussion

This is the first report of the successful establishment of a mixed-species honeybee colony, with individuals of *Apis cerana cerana* and *Apis mellifera ligustica* cohabiting, foraging and carrying out normal hive functions, for the greater part harmoniously, for over 50 days. Several cross-species interactions, such as dance following, trophallaxis and queen tending were observed during this period, indicating that ours was a normally functioning hive. We believe that this is an important breakthrough in the study of honeybees, and that such mixed-species hives will open exciting new avenues of research into various aspects of this social insect's biology.

We studied details of the dance communication (dance angle, waggle duration and recruitment success) of *Acc* and *Aml* in the mixed-species hive. The dance angles were not significantly different between *Acc* and *Aml* in the mixed-species hive, which means that both dance dialects indicated the same food source direction. However, the distance-dependent waggle durations were significantly different between *Acc* and *Aml* honeybees, regardless of whether they were in a pure colony or the mixed-species colony. The dialect differences of honeybee species are therefore encoded in the difference in waggle duration. Environmental variables, such as wind velocity, temperature and the surrounding landscape can be ruled out, as all bees were made to forage along the same flight path, and all dances for a given experiment, whose waggle durations were analysed, were recorded within a short period of time.

An early study on dance communication between two subspecies of *Apis mellifera* in a mixed hive similar to ours commented on the ‘misunderstandings’ that occur when workers of one subspecies follow the dancers of another [16]. However, the foragers in that study were still able to locate feeders in the vicinity of the food source, even after having followed dances in a different dialect. While the subspecies of *Apis mellifera* may have diverged around 0.67 million years ago [30], [31], our study confirms that the ability to use the information encoded in an unfamiliar dance extends even across species separated by six to eight million years of evolution [32], [33]. The *Acc* bees in our mixed-species colony were almost as successful as the *Aml* bees in locating a feeder advertised by more experienced *Aml* dancers, as indicated by our accuracy experiments. The *Acc* bees following *Aml* dances were not only recruited to the experimental feeder, but also preferentially chose the one at the correct distance (as indicated by *Aml* dances), when two unscented dummy feeders were presented at nearer and farther locations. These results highlight the highly conserved nature of not only the dance itself, but also the mechanisms by which the dance is interpreted by follower bees.

In the accuracy experiments, we also noticed that more recruited foragers, regardless of species, visited the nearer feeder at 400 m than the farther one at 600 m. This indicates that some of the recruited foragers perceived the exact directional, but approximate distance, information regarding the rewarded feeder site, and then searched for their final location after exploring the nearer feeder at 400 m. When comparing the visit frequency of foragers recruited by the same species or by a different species, we find that the number of recruited foragers in *intra*specific recruitment is always greater than that of *inter*specific recruitment, although not in a statistically significant manner.

Honeybee waggle dancers produce and release behaviourally active chemicals, which attract new foragers to follow dancers, and excite the followers to fly out of the hive [34]. From the results of dance-following behaviour in the mixed-species colony, we hypothesise that both *Acc* and *Aml* dancers might produce active chemicals to attract new *Acc* and *Aml* foragers, although new *Acc* foragers seem more sensitive to the active chemical than do *Aml* foragers. This is consistent with our observation that *Acc* honeybees are more likely to follow dances (in absolute terms) than *Aml* honeybees. Social learning is classically defined as “learning that is influenced by observation of, or interaction with, another animal (typically a conspecific) or its products” [35]–[39]. The possible interspecific chemical communication and dance-related interactions observed between *Acc* and *Aml* workers in the mixed-species colony are indicative of just this kind of social learning.

There has been a considerable amount of research on the sensory basis of the dance language [4], [5], [40], [41]. Although progress has been made recently in elucidating the underlying neural mechanisms of these remarkable abilities [42]–[45], the honeybee dance is still rarely discussed in the context of social learning [39]. Social learning might well be the mechanism by which bees of one species come to decode the dances of another, and locate the indicated food source; further studies will be required to investigate this possibility. For instance, do *Acc* bees become more proficient at decoding *Aml* dances with increasing foraging experience? In other words, do the kinds of ‘misunderstandings’ reported by Boch [16] manifest themselves early in a mixed-species colony's life, and then gradually disappear with time? If so, does the rate of improvement in ‘fluency’ in another species' code differ significantly from any ontogenetic improvements in the fluency in the code of one's own species for bees in a ‘pure’ colony? Particularly exciting is the possibility that naïve *Acc* bees, whose dances contain longer waggle runs, might, after a period of time, learn to perform shorter dances after following only *Aml* dances, and searching for the advertised resources. The mixed-species colony used in the present study has paved the way for investigating such questions relating to the learning component of the dance language.

We now know that honeybees have a variety of impressive cognitive skills and an amazing learning ability [39], [46]–[51]. Owing to the small brain size of the subjects, the study of honeybee learning has a good tradition of deconstructing seemingly complex phenomena, and explaining them in terms of simple processes. This provides an ideal perspective to study the mechanisms of social learning, too. The mixed-species colonies of *Acc* and *Aml* have paved a new way to study communication and learning between individuals of different species, which will be helpful in understanding the neural mechanisms of the striking dance language of honeybees.

## Materials and Methods

### Bees and the organization of mixed-species colony

Six colonies of *Apis mellifera ligustica* (4–6 frames) and *Apis cerana cerana* (3–4 frames) were kept at the apiary of Huajiachi Campus, Zhejiang University, Hangzhou, China.

We organized three mixed colonies consisting of an *Apis cerana cerana* (*Acc*) queen, *Acc* workers and *Apis mellifera ligustica* (*Aml*) workers (Figure 1), and two other mixed colonies consisting of an *Aml* queen, *Aml* workers and *Acc* workers. In the former colonies, the workers cohabited well for more than 20 days, while in the latter colonies, the *Acc* workers were killed and cleaned up by *Aml* workers after 2–3 days. Thus, we were only able to use the former mixed-species colony to carry out our experiments. We put the mixed colonies into observation hives after transporting them to the experimental location.

### Experimental site and standard training procedure

We set up two mixed honeybee colonies and transported them to the Agricultural School of Zhangzhou, Fujian province, China. The mixed colonies were put into an observation hive consisting of an *Acc* queen and equal numbers (1500 individuals) of *Aml* and *Acc* workers. In order to investigate whether *Acc* foragers in a mixed-species colony could be recruited by *Aml* dancers to a particular feeder, we designed two experiments. Firstly, eight to ten *Aml* workers were trained and marked to collect sugar syrup from an artificial feeder placed at different distances (50, 100, 150 and 200 m away) in a south-west direction from the observation hive on the campus of Agricultural School of Zhangzhou. A hive containing only *Acc* bees was placed five metres away from the mixed hive as a control for the possibility that stray *Acc* foragers might accidentally find the feeder during their normal foraging activities. When an *Acc* forager landed at the feeder, it was marked on the thorax with a paint mark; its appearance back at the experimental hive ensured that it came from the mixed-species colony. In this manner, we were able to confirm whether all *Acc* bees spotted at the feeder were from the mixed-species colony or not. In addition, any marked *Acc* forager arriving at the feeder a second time was caught and held for the duration of the experiment. This ensured that all the dances advertising the feeder location at the experimental hive were performed by *Aml* dancers.

In a second experiment, the observation hive was equipped with a moveable glass cover. Another mixed-species colony was put into this observation hive, and only *Aml* workers were trained and marked to collect sugar syrup from an artificial feeder placed at different distances (50, 100, 150 or 200 m away) in a south-west direction from the observation hive. We were able to mark *Acc* workers, who were following *Aml* dances, on the thorax and abdomen with paint. Then we monitored the feeder, where we caught any newly-recruited *Acc* foragers, and held them in a bottle for the duration of the experiment.

### Accuracy of recruitment between *Aml* and *Acc*

**Figure 6:**
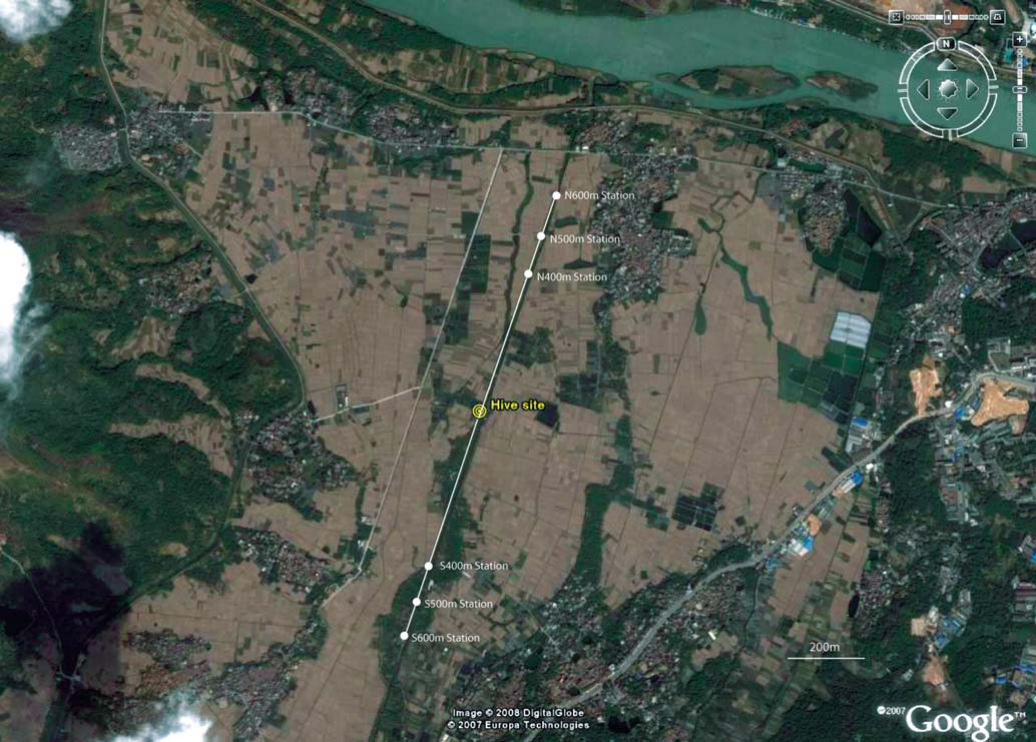
Aerial Photograph of Experimental Site. The experiments were carried out on the banks of the Da-Mei-canal in Zhangzhou, Fujian province, China. In the first experiment, three identical feeder-stations were located along the Da-Mei canal, in the south-west direction from the hive, at 400 m, 500 m and 600 m respectively. In the second experiment, three identical feeder-stations were located along the Da-Mei canal, in the north-east direction from the hive, at 400 m, 500 m and 600 m respectively (The photo was downloaded from Google earth).

During December 2007 to February 2008 we carried out complementary experiments to assess the accuracy of *Acc* foragers recruited by *Aml* dancers and vice versa. We set up an observation hive with a mixed-species colony of an *Acc* queen, *Acc* workers and *Aml* workers on the banks of the Da-Mei-canal in Zhangzhou, Fujian province, China (Figure 6). In the first experiment, three identical, unscented feeder-stations [9] were located along the Da-Mei canal, in the south-west direction from the hive, at 400 m, 500 m and 600 m respectively. Then, more than 50 *Aml* foragers were collected from the entrance of the observation hive, and directly carried to the 500 m feeder-station to collect sugar syrup (2.0 M sugar). Only *Aml* foragers were trained to collect sugar syrup at the 500 m feeder. After three days' training, around 13 *Aml* foragers had learnt to visit the 500 m station regularly. They were marked with paint, and after a few visits, they started to dance to recruit other foragers at the mixed-species colony. In the tests, the previously used feeders were replaced with fresh unscented feeders. The feeders at the 400 m and 600 m stations were unrewarded. The visit frequency of the newly-recruited foragers was monitored at each feeder-station from 9:00 a.m. to 12:00 a.m., and from 12:30 p.m. to 16:30 p.m.

We monitored the feeders at all three positions, noting the presence of new foragers at the feeders. To be sure that all new foragers came from our experimental hive, any new recruits arriving at the 500 m feeder was marked with a unique colour combination. All such bees were seen to arrive at the observation hive. We only counted the newly recruited foragers. When the marked recruited foragers visited the 500 m feeder, we did not count them again.

### Waggle duration comparison

To study the effect of a mixed-species colony on the waggle dance, we set up an observation hive with a mixed-species colony of an *Acc* queen, *Acc* workers and *Aml* workers on the banks of the Da-Mei-canal in Zhangzhou, Fujian province, China. The road that ran along one bank of the canal was mostly deserted. The weather was warm enough for foragers to collect syrup, and the natural food sources were limited. The artificial feeders were set in the south direction at 100 m, 200 m, 300 m and 400 m away from the observation hive, respectively. We carried out the experiments from 9:00 a.m. to 12:00 p.m., and from 12:30 p.m. to 16:30 p.m. During these experiments, the weather was sunny (we stopped the experiments during times of heavy cloud cover and rain), and the air temperature ranged between 18–25° Celsius. We made digital video recordings, at the observation hive, of the dances of *Acc* and *Aml* foragers who had been marked at the feeder. As controls, we also recorded the dances of foragers from single-species *Acc* and *Aml* colonies, who had been trained to feeders at equivalent distances along the same flight path as the mixed-species colony. The waggle durations of at least ten dancers, with five foraging trips per individual from each species, were recorded and analysed using Ulead VideoStudio 9.0 SE DVD software (http://www.ulead.com/vs/) for feeder distances of 100 m, 200 m, 300 m and 400 m.

### Dance angle comparison

The dance angle, corresponding to the angle between the sun's azimuth and the indicated food source outside the hive, is the direction of the waggle run relative to the direction of gravity. Ten pairs of *Acc* and *Aml* dancers were analysed in the digital video records using Ulead VideoStudio 9.0 SE DVD software (http://www.ulead.com/vs/). Five waggle phases of each individual were measured, and the average was regarded as the dance angle. To ensure the comparability of dances from each species, we recorded and analysed pairs of individual dances occurring within ten minutes of each other for feeder distances of 100 m, 200 m, 300 m and 400 m.

### *Acc* and *Aml* workers following dancers

When we analysed the waggle duration data of the dancers, we also counted the number of *Acc* and *Aml* workers following a dancer in the pure and mixed-species colonies. The number of workers following at least ten dancers from each species was recorded and analysed for feeder distances of 100 m, 200 m, 300 m, 400 m and 500 m.

### Statistical analysis

The dances were evaluated as follows. For each dance, the mean waggle duration was estimated by averaging the waggle durations over all loops. Then, the mean waggle duration for each bee was obtained by averaging the mean waggle durations over all of its dances at that feeder position. Finally, the mean waggle duration of all bees was calculated from the mean waggle durations for the individuals. The standard error of the mean was also calculated and displayed in the graphs.

Linear regressions of the data were computed using the GraphPad Prism (GraphPad Software, San Diego, California, United States) statistical analysis package. To compare waggle durations and regression slopes of different data sets, we used the same statistical package, which implemented the slope comparison test described in Sokal and Rohlf (1995) [52].

To compare waggle durations of *Apis mellifera* and *Apis cerana* in the pure colony and the mixed-species colony, we used Analysis of Variance for Two-stage Nested Design of DPS Software (http://www.chinadps.net/index.htm). To compare the visit frequencies of recruited *Acc* and *Aml* at 400 m, 500 m, and 600 m positions, we used a One-way ANOVA and Tukey's test of DPS Software (http://www.chinadps.net/index.htm). We used a t-test with Welch correction to compare the number of *Acc* and *Aml* bees following *Acc* and *Aml* dancers in the mixed-species colony. To compare the dance angles collected from *Acc* dancers with those of *Aml* dancers, we used a pairwise t-test of DPS software [53].

## Supporting Information

Table S1 A summary of statistical significant tests to compare waggle duration.
(0.05 MB DOC)

Figure S1 Accuracy of recruitment by Acc dancers. The experiments were carried out on the banks of the Da-Mei-canal in Zhangzhou, Fujian province of China (see Figure 4). Similar to the experiments in which Acc and Aml foragers were recruited by Aml dancers, in this experiment, only Acc foragers were trained to collect sugar syrup at the 500 m feeder in the north-east direction from the hive,. After three days' training, average 9 Acc foragers had learnt to visit the 500 m station regularly. In the tests, the previously used feeders were replaced with fresh unscented feeders. The feeders at the 400 m and 600 m stations were unrewarded. Figure S1a shows that the visit frequency of Acc foragers recruited by Acc dancers is significantly different at 400 m, 500 m and 600 m (F2,30 = 5.89, p = 0.007, One-way ANOVA), and that the frequency of recruited Acc at 500 m was greater than that at 400 m (P = 0.0175, Tukey's test) and 600 m (P = 0.0138, Tukey's test). Comparing visit frequencies at 400 m and 600 m, more bees visited the 400 m feeder than the 600 m feeder, but the difference was not significant (p = 0.9949, Tukey's test). Figure S1b shows a similar trend for Aml foragers recruited by Acc dancers. The visit frequency of recruited Aml was significantly different at 400 m, 500 m and 600 m (F2,30 = 10.207, p = 0.0004, One-way ANOVA), and the frequency of recruited Aml at 500 m was greater than that at 400 m (P = 0.0045, Tukey's test) and 600 m (P = 0.0006, Tukey's test). Comparing visit frequencies at 400 m and 600 m, more bees visited the 400 m feeder than the 600 m feeder, but the difference was not significant (p = 0.7378, Tukey's test). Comparison of Acc foragers and Aml foragers recruited by Acc dancers showed that Acc dancers can recruit more Acc foragers to the 500 m feeder than Aml foragers, but the difference is not significant (F1,66 = 0.8702, p = 0.4037, ANOVA for Two-stage Nested Design).
(0.97 MB TIF)

Movie S1 An Acc queen lays an egg while being attended to by lighter-coloured Aml workers.
(9.96 MB MPG)

Movie S2 Apis cerana cerana (Acc) bees (dark abdomens) following the dance of a marked and an unmarked Apis mellifera ligustica (Aml) forager (lighter abdomens) in the mixed-species colony. Both dancers had been trained to an artificial feeder 200 m away from the hive.
(9.11 MB MPG)

Movie S3 Aml and Acc bees following the dance of a marked Acc forager in the mixed-species colony. Like the bees in video S1, this dancer had also been trained to the same 200 m position.
(9.02 MB MPG)

Movie S4 Trophallaxis between a marked Acc worker (right) and an unmarked Aml worker (left).
(5.77 MB MPG)
