# Supplementary material for: East Learns from West: Asiatic Honeybees Can Understand Dance Language of European Honeybees
Source: PLoS One. 2008 Jun 4;3(6):e2365. doi: 10.1371/journal.pone.0002365 (PMC2391287; doi:10.1371/journal.pone.0002365)
Supplement: Table S1 — A summary of statistical significant tests to compare waggle duration. (0.05 MB DOC) [file pone.0002365.s001.doc]

Table S1: Summary of statistical significant tests on waggle dance durations

(Analysis of Variance for Two-stage Nested Design and Tukey’s test of DPS Software)

| **100M**  *F_3,200_*=20.3069, p=0.0001  Tukey05=78.1650　Tukey01=97.0592 | | | | | |
| --- | --- | --- | --- | --- | --- |
| No. | Mean | 1 | 2 | 3 | 4 |
| 1 | 412.8333 |  | 0.2012 | 0.0012 | 0.0011 |
| 2 | 354.3333 | 58.5 |  | 0.1775 | 0.1647 |
| 3 | 293.8333 | 119 | 60.5 |  | 0.9999 |
| 4 | 292.6667 | 120.1667 | 61.6667 | 1.1667 |  |
| **200M**  *F_3,210_*=7.8133, p=0.0004  Tukey05=111.0102　Tukey01=137.6612 | | | | | |
| No. | Mean | 1 | 2 | 4 | 3 |
| 1 | 690.8333 |  | 0.9199 | 0.0001 | 0.0001 |
| 2 | 664.5758 | 26.2576 |  | 0.0003 | 0.0001 |
| 4 | 475.8182 | 215.0152 | 188.7576 |  | 0.6446 |
| 3 | 427.1667 | 263.6667 | 237.4091 | 48.6515 |  |
| **300M**  *F_3,215_*=49.3615, p=0.0001  Tukey05=131.1142　Tukey01=162.4925 | | | | | |
| No. | Mean | 1 | 2 | 4 | 3 |
| 1 | 982.3889 |  | 0.9392 | 0.0001 | 0.0001 |
| 2 | 954.3333 | 28.0556 |  | 0.0001 | 0.0001 |
| 4 | 601.8788 | 380.5101 | 352.4545 |  | 0.1798 |
| 3 | 498.5 | 483.8889 | 455.8333 | 103.3788 |  |
| **400M**  *F_3,220_*=28.162, p=0.0001  Tukey05=204.3656　Tukey01=253.1279 | | | | | |
| No. | Mean | 1 | 2 | 4 | 3 |
| 1 | 1259.561 |  | 0.9823 | 0.0001 | 0.0001 |
| 2 | 1231.242 | 28.3182 |  | 0.0001 | 0.0001 |
| 4 | 784.9091 | 474.6515 | 446.3333 |  | 0.8288 |
| 3 | 719.8939 | 539.6667 | 511.3485 | 65.0152 |  |

Note: 1. *Acc* in pure colony; 2. *Acc* in mixed colony; 3. *Aml* in mixed colony;

4. *Aml* in pure colony;
