# Supplementary figures and images for: East Learns from West: Asiatic Honeybees Can Understand Dance Language of European Honeybees
Source: PLoS One. 2008 Jun 4;3(6):e2365. doi: 10.1371/journal.pone.0002365 (PMC2391287; doi:10.1371/journal.pone.0002365)

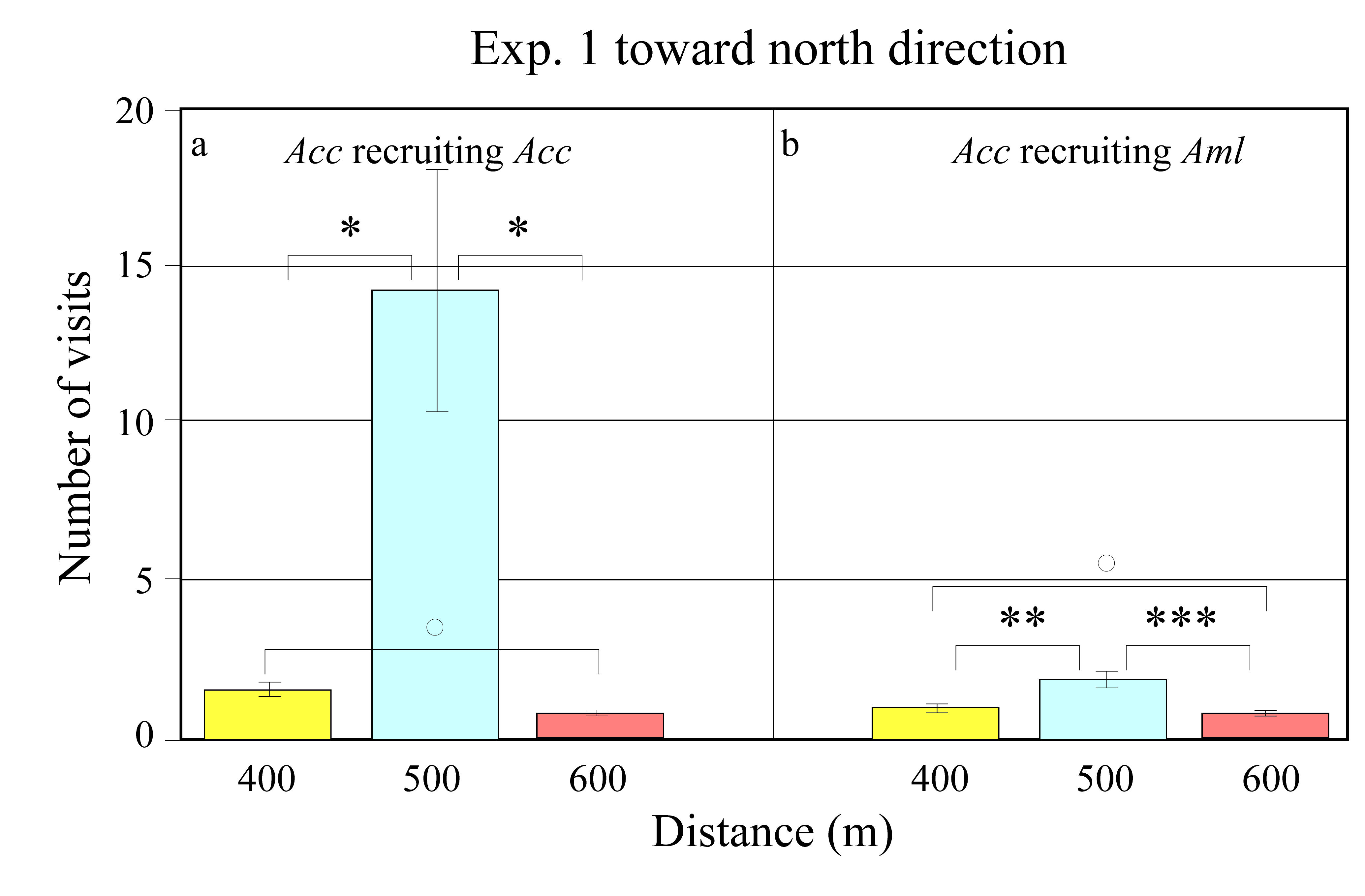

Supplement: Figure S1 — Accuracy of recruitment by Acc dancers. The experiments were carried out on the banks of the Da-Mei-canal in Zhangzhou, Fujian province of China (see Figure 4). Similar to the experiments in which Acc and Aml foragers were recruited by Aml dancers, in this experiment, only Acc foragers were trained to collect sugar syrup at the 500 m feeder in the north-east direction from the hive,. After three days' training, average 9 Acc foragers had learnt to visit the 500 m station regularly. In the tests, the previously used feeders were replaced with fresh unscented feeders. The feeders at the 400 m and 600 m stations were unrewarded. Figure S1a shows that the visit frequency of Acc foragers recruited by Acc dancers is significantly different at 400 m, 500 m and 600 m (F2,30 = 5.89, p = 0.007, One-way ANOVA), and that the frequency of recruited Acc at 500 m was greater than that at 400 m (P = 0.0175, Tukey's test) and 600 m (P = 0.0138, Tukey's test). Comparing visit frequencies at 400 m and 600 m, more bees visited the 400 m feeder than the 600 m feeder, but the difference was not significant (p = 0.9949, Tukey's test). Figure S1b shows a similar trend for Aml foragers recruited by Acc dancers. The visit frequency of recruited Aml was significantly different at 400 m, 500 m and 600 m (F2,30 = 10.207, p = 0.0004, One-way ANOVA), and the frequency of recruited Aml at 500 m was greater than that at 400 m (P = 0.0045, Tukey's test) and 600 m (P = 0.0006, Tukey's test). Comparing visit frequencies at 400 m and 600 m, more bees visited the 400 m feeder than the 600 m feeder, but the difference was not significant (p = 0.7378, Tukey's test). Comparison of Acc foragers and Aml foragers recruited by Acc dancers showed that Acc dancers can recruit more Acc foragers to the 500 m feeder than Aml foragers, but the difference is not significant (F1,66 = 0.8702, p = 0.4037, ANOVA for Two-stage Nested Design). (0.97 MB TIF) [file pone.0002365.s002.tif]
